# Fetomaternal Outcome Among the Pregnant Women Subject to the Induction of Labor

**DOI:** 10.7759/cureus.15216

**Published:** 2021-05-24

**Authors:** Sarah Kazi, Uroosa Naz, Urooj Naz, Aruna Hira, Aneela Habib, Fouzia Perveen

**Affiliations:** 1 Obstetrics and Gynecology, Civil Hospital, Karachi, PAK; 2 Obstetrics and Gynecology, Dow University of Health Sciences (DUHS), Karachi, PAK; 3 Gynecology and Obstetrics, Civil Hospital, Karachi, PAK

**Keywords:** induction of labour, feto-maternal outcome, mode of delivery

## Abstract

Introduction

Induction of labor (IOL) is characterized by stimulating contractions of the uterus just before the instantaneous onset of labor, with or without amniotomy. According to the recommendation of the World Health Organization (WHO), induction must only be carried out when there is a clear medical need for one and when potential benefits outweigh the expected harm that may be caused by it. The present study was to determine the frequency of fetomaternal outcomes among pregnant women subject to the induction of labor.

Methods

The present prospective cross-sectional study was conducted over a period of one year starting from June 17, 2018, to July 25, 2019, in the Department of Obstetrics and Gynecology Unit III, Civil Hospital Karachi. After institutional ethical committee approval, 302 pregnant women who were subject to induction of labor were enrolled using a non-probability consecutive sampling technique. Outcome variables, i.e., postpartum hemorrhage, mode of delivery, hospital stay more than seven days, birth asphyxia, Apgar score < 7 at five minutes, neonatal jaundice, and low birth weight were noted. IBM Statistical Package for Social Sciences (SPSS) Statistics for Windows, version 21.0 (IBM Corp., Armonk, NY) was used for data analysis.

Results

A total of 302 women with an average age range was 18-45 years with a mean age of 28.5 ± 4.47, body mass index (BMI) 29.83 ± 3.83, and mean gestational age was 37 ± 4.3. Almost 205 (67.9%) of the cases were booked. One hundred and eighty (59.6%) were nulliparas, 57(18.8%) had para-1, 43 (14.4%) had para-2, and 22 (7.14%) had par-3. When fetomaternal outcome among the pregnant women subject to induction of labor was observed, postpartum hemorrhage was observed in 55 (18.21%), hospital stay more than seven days was in 51 (17%), birth asphyxia was in 45 (14.9%), neonatal jaundice was in 53 (17.6%), low birth weight was in 15 (4.96%), Apgar score < 7 was in 48 (16%), 39 (13%) women underwent for C-section and 263 (87%) of the women delivered vaginally.

Conclusion

This study concludes that the induction of labor (IOL) is safe and reliable and less risk of adverse feto-maternal outcome is associated with pregnancies between 37 weeks and 42 weeks of gestation. The evidence regarding IOL prior to 37 weeks and beyond 42 weeks of gestation is inadequate to reach any conclusion.

## Introduction

Globally, induction of labor (IOL) has seen a gradual upward trend irrespective of the medical conditions, and today, it is used in approximately 25% of all the pregnancies in the developed world [1,2]. However, its advantages in term pregnancies have mostly been one of the controversies [3]. On the other hand, with regards to post-term pregnancies, IOL has proven to result in better maternal and fetal outcomes [4].

It is not uncommon for induction to be carried out for social or location reasons without a proper medical indication requiring its use [2]. Regardless, a number of carefully planned studies have been carried out to evaluate induction for these various medical indications [5]. When carried out with success, induction often results in delivery through the vagina, but at times it does not go according to plan with the following potential risks like cesarean section birth. An increase in the rate of operative vaginal delivery with excessive activity of the uterus and abnormal patterns of the fetal heart rate and improper estimation of delivery rate may result in preterm delivery of the infants and possible cord [1,2,5].

The primary indicator of the chances of success is the state of the cervix. A cervix that is not ripe increases the likelihood of a non-vaginal delivery [6]. Given that there is an increased progression in perinatal mortality and fetal compromise with gestation extending over 37 weeks, it is during weeks 37 and 41 that induction of labor has the massive potential to better neonatal outcomes [7]. A systematic review carried out recently compared the policy of labor induction of all types of pregnancies with expected management showed that induction is often associated with a reduced number of fetomaternal mortalities and morbidities [1].

In the correct estimation of the delivery date is the leading reason for pregnancies to get prolonged their normal times [3,4]. Maternal genetic factors, previous history of incorrect dates, obesity, and the male gender of the fetus are some of the risk factors associated with induction [8]. The criteria for evaluating postdates include the correlation of menstrual history, ultrasonography (USG), and other medical findings. In early pregnancies, dating from the ultrasonograph may help in improving the accuracy of estimated date of delivery (EDD) [9].

A study carried out on 1000 pregnant women undergoing induction of labor in India found that one in three of the total had to go through a cesarian section and around 70% of the pregnancies were delivered through the vagina. Twenty percent of the total women spent more than seven days in the hospital. Birth asphyxia was observed in 17% of the children, around 20% of the newborns suffered from jaundice, and 5% had a relatively low birth weight [10,11].

IOL is commonly practiced in Obstetrics and there is always a need to know more about its safety. This study fits perfectly in the existing literature, where outcomes of IOL were studied. Such studies definitely help in keeping consensus and need timely review in the light of updated literature.

The primary goal of this study is to determine the prevalence of fetomaternal outcomes in pregnant women who are subjected to the induction of labor. However, there is a dearth of studies and overall research on this topic in Pakistan. Internationally, there is a substantive amount of literature on IOL and its outcomes, but only a few outcomes have been observed and the prevalence of the observed outcomes was also different. Different demographic features including literacy rate, socioeconomic status, and ethnicity may be the reason for this disparity in outcomes. Through my study, data will be provided and local guidelines will be developed in order to develop a proper strategy according to the results.

## Materials and methods

The observational study was conducted from the duration of the period between June 17, 2018, and July 25, 2019, in the Department of Obstetrics and Gynecology Unit III Civil Hospital Karachi. After institutional ethical committee approval, 302 pregnant women who were subject to induction of labor were enrolled using a non-probability consecutive sampling technique. All women of any parity, age 18-45 years among the pregnant women subject to induction of labor. Women with a gestational age of 37 weeks to 42 weeks confirmed on first-trimester ultrasound. The exclusion criteria were pregnancy with complications like diabetes mellitus, renal pathology, cardiac disease. Those women uterine surgeries like myomectomy, malpresentation, post-term pregnancies, hysterotomy, or any other uterine scar and pelvic structure deformities. Informed consent of the study population with the assurance to keep their information confidential their demographics were obtained to include in the study. Outcome variables, i.e., postpartum hemorrhage, mode of delivery, hospital stay more than seven days, birth asphyxia, Apgar score < 7 at five minutes, neonatal jaundice, low birth weight (LBW) were noted. The relevant effect modifiers/confounders like age, gestational age, parity, socioeconomic status, educational status, booking status, and body mass index (BMI) were analyzed. The biasness was controlled by strictly following the inclusion and exclusion criteria. Confidentiality of the patient was maintained.

Data were analyzed by IBM Statistical Package for Social Sciences (SPSS) Statistics for Windows, version 21.0 (IBM Corp., Armonk, NY). Mean and the standard deviation was calculated for age, gestational age, height, weight, and BMI. Frequency and percentage were calculated for socio-economic status, educational status, parity, booking status, and outcome variables, i.e., postpartum hemorrhage, mode of delivery, hospital stay more than seven days, birth asphyxia, Apgar score < 7 at five minutes, neonatal jaundice, low birth weight (LBW). Stratification with respect to age, gestational age, parity, socioeconomic status, educational status, booking status, and BMI was done. The post-stratification chi-square test was applied. A p-value less than equal to 0.05 was taken as significant.

## Results

The age range of the mothers in this study was 18-45 years with mean age 28.5 ± 4.47 ranging between 16 years and 45 years, with mean height 160 ± 8.09, mean weight 69.51 ± 8.91, mean BMI 29.83 ± 3.83, and mean gestational age was 37± 4.3. Almost 205 (67.9%) of the cases were booked and 97 (32.1%) of the cases were un-booked. Out of 302 women, 180 (59.6%) were nulliparas, 57 (18.8%) had para-1, 43 (14.4%) had para-2, and 22 (7.14%) had par-3. The distribution of income level was stratified and found that 53 (17.5%) had monthly income less than and equal to PKR 20,000, 141 (46.66%) had monthly income between PKR 21,000 and 50,000, and 108 (35.83%) had monthly income greater than PKR 50,000. Out of 302 women, 46 (15.3%) were illiterate, p8 (32.5%) had primary education, 83 (27.5%) had secondary education, 45 (14.8%) were intermediate and 30 (9.84%) had education till graduation or above. When fetomaternal outcome among the pregnant women subject to induction of labor was observed, postpartum hemorrhage was observed in 55 (18.21%), hospital stay more than seven days was in 51 (17%), birth asphyxia was in 45 (14.9%), neonatal jaundice was in 53 (17.6%), low birth weight was in 15 (4.96%), Apgar score < 7 was in 48 (16%), 39 (13%) women underwent for C-section and 263 (87%) of the women delivered vaginally. When outcome variables were stratified with respect to age, gestational age, BMI, parity, booking status, educational status, and income status, no significant difference was observed. The descriptive characteristics of the pregnant women are summarized in Table 1.

**Table 1:** Baseline and descriptive characteristics of the pregnant women

Baseline and Clinical Characteristics	Frequency	Percentage
Booking status		
Booked	205	67.90%
Un-booked	97	32.10%
Parity distribution		
Nullipara	180	59.60%
Para -1	57	18.80%
Para -2	43	14.40%
Para > 3	22	7.14%
Socioeconomic status		
Less than equal to Rs. 20,000	53	17.50%
Between Rs. 21,000 and 50,000	141	46.68%
Greater than Rs. 50,000	108	35.57%
Educational status of mother		
Illiterate	46	15.30%
Primary	98	32.50%
Secondary	83	27.50%
Intermediate	45	14.86%
Graduate and above	30	9.84%
Fetomaternal outcomes among the pregnant		
Postpartum hemorrhage	55	18.21%
Hospital stay > 7 days	51	17.00%
Birth asphyxia	45	14.90%
Neonatal jaundice	53	17.60%
Low birth weight	15	4.96%
Apgar score < 7 at 5 min	48	16.00%
Mode of Delivery		
Cesarean delivery	39	13.00%
Vaginal delivery	263	87.00%

Table 2 shows a comparison of fetomaternal outcomes in different parameters of the study. Age group is the only variable which shows the significant difference (P-value 0.006) in comparison of pregnancy vaginal pH where the majority of participants with the age group of 18 years to 30 years fall in this age accounted for 43 (78.2%) with (odds ratio {OR}: 2.5 95% 1.28 to 5.1), women who are between 18 years and 30 years were more likely stayed more in hospital as compared to 30-45 years (P<0.001), (OR: 5.75 2.37 to 13.98). Also observed that low age groups were significantly delivered low birth weight of the baby (P=0.043). Furthermore, most of the lower age groups were significantly delivered with C-sections and found that 3.15 times more likely lower age pregnancies delivered through vaginally cesarean section.

**Table 2:** Comparison of fetomaternal outcome among the pregnant women with age groups pH = potential of hydrogen, LBW = low birth weight, OR = odds ratio, LL = lower limit, UL = upper limit *Significant at 5%.

	Age Group			
	18-30	30-45	OR (95% LL- UL)	P-value
pH	43 (78.2%)	12 (21.8%)	2.5 (1.28-5.1)	0.006
Hospital stay	45 (88.24% )	6 (11.76% )	5.75 (2.37-13.98)	<0.001*
Birth asphyxia	35 (77.78% )	10 (22.22% )	2.41 (1.14-5.09)	0.018
LBW	13 (86.67% )	2 (13.33% )	0.237 (0.052-1.07)	0.043
Mode of delivery	32 (82.05% )	7 (17.95% )	3.15 (1.34-7.41)	0.006

## Discussion

Globally, IOL has seen a gradual upward trend irrespective of the medical conditions, and today, it is used in approximately 25% of all the pregnancies in the developed world [2]. However, its advantages in term pregnancies have mostly been one of controversy [9]. On the other hand, with regards to post-term pregnancies, IOL has proven to better maternal and fetal outcomes [12]. A large number of studies on the subject show similar results regarding lower chances of lower segment cesarean section (LSCS) in the group who undergo labor induction [13]. On the other hand, similar studies have identified induction of labor as a contributing reason for the increased rate of deliveries through the caesarian section [14]. For the purposes of our study, the percentage of pregnancies undergoing the cesarean is documented to be around 15% [3,15]. A detailed analysis using metadata provides evidence for a lower rate using the cesarean section in comparison to the 18% overall institutional rate during the time duration of our study [16].

In this study, we observed that postpartum hemorrhage was found in 55 (18.21%), hospital stay more than seven days was in 51 (17%), birth asphyxia was in 45 (14.9%), neonatal jaundice was in 53 (17.6%), low birth weight was in 15 (4.96%), Apgar score < 7 was in 48 (16%), and 39 (13%) women underwent for C-section and 263 (87%) of the women delivered vaginally [17]. Whereas, in this study, we have excluded the patients having any of the above-mentioned complications. Some studies revealed that the induction of labor has been identified as contributing factor to the rising rate of cesarean deliveries [18]. The cesarean section rate following induction in this study was 13%. Ameta-analysis supports this low cesarean section rate compared to the overall institutional rate of 18% during the study period. In the meta-analysis [18,19]

A total of 302 women having uterine rupture were enrolled in this study. The mean age was 31.1± 8.8, the mode was 39, and the median was 31 with ranging between 16 years and 45 years. Similar findings were reported in previous studies done by Latika, and Omole-Ohonsi, and Attah, respectively [20,21].

Regarding the parity, the majority of patients 72 (49.3%) in this study were multigravidas, followed by 70 (47.9%) in the parity group of primigravida, only four cases having to follow on multigravida. A similar result was found in the study done by Malik where 42.71% of women were paras 2-4 [22].

In our study, the prevalence of a low Apgar score was 15% compared to 10% in a different similar study [23]. The occurrence of jaundice in the neonatal stage was documented to be around 20%. The elevated percentage of jaundice may be a direct result of forceps delivery having a higher incidence [24]. Another study documented the percentage of neonatal jaundice to be as high as 40% while a meager 2% was accounted for hypoglycemia and an even less 1% accounting for birth asphyxia [25]. A different study revealed increased changes of Apgar being less than seven, extremely less weight at birth, admission into the neonatal intensive care unit (NICU), and belated breastfeeding linked with labor induction [24].

This study documented the rate of induction to be around 12% which was over the rate documented from Nigeria and an average of 5% reported by a study carried out by Bukola et al. but the rate was within the range as documented in the U.S to be between 10% and 35% [26]. The most common medical indicator for induction was found to be postdate accounting for 45% of the total cases, being very similar in percentage terms to those reported in Maiduguri, Nigeria [27].

In this study, the combined success rate for induction was around 75% which is very identical to the figure of 70% being documented in the U.S but much less than a 90% success rate being documented by Ekele et al. in Sokoto, Nigeria [28]. Nevertheless, the rate of success of around 75% in comparison to 40% for misoprostol and catheter, respectively, was much less than the finding in Noor et al. [29].

For the purposes of our study, only short-term feto-maternal outcomes were taken into consideration disregarding the outcomes of the long-term nature. However, given that a similar study had not been carried out, so there was no point of reference or comparison. The type of sampling technique employed was non-probability consecutive which would not allow the results to be generalized across the board. Some of the studies have also shown poor feto-maternal outcomes. Thus more studies with the control group should also be carried out.

## Conclusions

According to this study, IOL is found to be particularly useful and effective in pregnancies of a high-risk nature when the gains through early delivery outweigh the risks associated with continuation. However, on the flip side, there are chances for the attendant to face problems with a risk of failure but these risks can substantially be reduced with careful selection of the patients and proper monitoring and evaluation of the fetus to make sure that the final outcome results in a healthy baby and mother. 
